# Recursive Feature Selection with Significant Variables of Support Vectors

**DOI:** 10.1155/2012/712542

**Published:** 2012-08-15

**Authors:** Chen-An Tsai, Chien-Hsun Huang, Ching-Wei Chang, Chun-Houh Chen

**Affiliations:** ^1^Department of Agronomy, National Taiwan University, Taipei 106, Taiwan; ^2^Department of Statistics, Columbia University, New York, NY 10027, USA; ^3^Division of Personalized Nutrition and Medicine, National Center for Toxicological Research, Food and Drug Administration, Jefferson, AR 72079, USA; ^4^Institute of Statistical Science, Academia Sinica, 128 Academia Road, Section 2, Taipei 115, Taiwan

## Abstract

The development of DNA microarray makes researchers screen thousands of genes simultaneously and it also helps determine high- and low-expression level genes in normal and disease tissues. Selecting relevant genes for cancer classification is an important issue. Most of the gene selection methods use univariate ranking criteria and arbitrarily choose a threshold to choose genes. However, the parameter setting may not be compatible to the selected classification algorithms. In this paper, we propose a new gene selection method (SVM-*t*) based on the use of *t-*statistics embedded in support vector machine. We compared the performance to two similar SVM-based methods: SVM recursive feature elimination (SVMRFE) and recursive support vector machine (RSVM). The three methods were compared based on extensive simulation experiments and analyses of two published microarray datasets. In the simulation experiments, we found that the proposed method is more robust in selecting informative genes than SVMRFE and RSVM and capable to attain good classification performance when the variations of informative and noninformative genes are different. In the analysis of two microarray datasets, the proposed method yields better performance in identifying fewer genes with good prediction accuracy, compared to SVMRFE and RSVM.

## 1. Introduction

Feature selection is a critical issue for disease subtyping and cancer classification. There are two goals when optimizing classification procedures, attaining highest accuracy and selecting smallest set of features. With the development of microarray technology, experimenters can detect expression profiles for more than ten thousands gene at a time. Classification problem with such huge amount of genes lead to inefficiency, inconsistency, and bias. There are many discussions on reducing the features by univariate rankings of gene and selecting the genes with highest rankings to build a classifier, for example, *t*-statistics (Golub et al. [1], Furey et al. [2], and Li et al. [3]) and *F*-score (Chen and Lin [4]) related ranking methods. Feature selection is the most challenging task in the pattern classification research especially for high-dimensional data.

Depending on the classification algorithms, feature selection techniques can be classified into three main groups: embedded, filter, and wrapper approaches. The filter methods rank features according to some criteria or indices of relevance which are completely independent of the classification algorithm such as *P* value. The filter approach is a stand-alone prior step, regardless of which classification algorithm will be used. Afterwards, the selected feature subset will be applied to the classification algorithm. The effects of the selected features on the performance of the algorithm are not taken into account. Classical classification algorithms, such as Fisher's linear discriminant analysis and *k*-nearest neighbour, often use the filter approach to select relevant predictors prior to classification (Chen and Lin [4], Roepman et al. [5], Mark and Kung [6], Pavlidis et al. [7], and Yu and Liu [8]). In the wrapper approach, the model selection algorithms are wrapped in the search process of feature subsets. This has the advantages that the feature selection process can take into account feature dependencies while building the classification model. However, these methods have high computational complexity to repeatedly training and testing predictors each time a new subset is defined. West et al. [9] used binary regression coefficients as importance in scoring genes to the contribution to the classification. Díaz-Uriarte and Alvarez de Andrés [10] proposed the use of Gini index as variable importance to perform gene selection in the classification algorithm of random forest. Sharma et al. [11] proposed a wrapper-like feature selection method based on null linear discriminant analysis (LDA) method. Embedded methods are an integral part of feature selection techniques and specific classification algorithms, for example, decision trees or neural networks with regularization. In addition, Sharma et al. [12] proposed a successive feature selection algorithm to iteratively eliminate redundant features with minimal information in terms of classification accuracy. Their method combines filters and wrappers together to search for the best top-r feature subset. A recent review (Saeys et al. [13]) summarized many more feature selection techniques and discussed their use for bioinformatics applications.

Over the recent years, support vector machine (SVM; Cristianini and Shawe-Taylor [14]), a supervised machine learning, is widely used in classification problem especially with high-dimensional data such as microarray gene expression profile. SVM maps input data points to construct maximal-marginal hyperplane in higher dimension space to classify data with different class labels. The hyperplane is constructed using only the support vectors (i.e., data that lie on the margin). The general form of the hyperplane is represented as

(1) f(x)=∑i=1nyiαiK(x,xi)+b,

where the training set is of size *n*, *x*_*i*_ is the input data of sample *i*  (*i* = 1,2,…, *n*), *α*_*i*_ is the Lagrange multiplier solved from the training set. *y*_*i*_ ∈ (−1, 1) is the class label for sample *i*, *K*(*x*, *x*_*i*_) is the kernel function, **x** is the variable vector of a sample, and *b* can be viewed as intercept. The optimized weight vector equals to ∑_*i*=1_^*n*^ *α*_*i*_ *y*_*i*_ *ϕ*(x_*i*_), where *α*_*i*_ is nonzero if *i* belongs to support vector, otherwise *α*_*i*_ equals to 0, and *ϕ*(·) is a nonlinear mapping function from input space to feature space.

Support vector machine has many applications and performs very well on microarray related classification problems (Furey et al. [2] and Brown et al. [15]). In addition, many gene selection methods based on SVM have been proposed previously. For example, Guyon et al. [16] proposed a support vector machine recursive feature elimination algorithm (SVMRFE), which uses the coefficients of weight vector to compute the feature ranking score. However, like the concept of slope, the selected genes with higher coefficients of weight means that they will have higher expression values compared to nonselected genes. Hence, some noisy but high-expression value genes have high possibility to be selected. On the other hand, Zhang et al. [17] proposed the recursive SVM feature selection (RSVM), which combines the weight and data information (i.e., class mean) to formulate selection criterion. Such method takes all data information into consideration and the outlier data is also included. However, such feature selection result is greatly affected by class label assignment. There is no general rule for assigning the positive and negative signs to the two classes. Hence, such unstable criterion is difficult to use. Hence, we propose a new feature selection criterion SVM-*t* based on the use of *t*-statistics embedded in support vector machine. We use the univariate ranking method on support vectors for identifying significant genes and the backward elimination procedure follows the workflow of RSVM with nested subsets of feature. The aim of combining procedures is to identify more significant genes among the nearest support vectors. In this paper, we compare the performance of the three SVM-based gene selection methods via extensive simulations and real microarray data analyses.

## 2. Materials and Methods

### 2.1. SVMRFE

In general, all classification problems can be generalized to two-class classification problem. If there are more than two-classes, the simplest and widely used approach is the multiple one-against-all scheme. Hence, multiple class problems can be reduced to multiple simple two-class problem. Consider a binary decision function with linear kernel, the function in (1) can be represented as:

(2) sgn⁡(f(x))=sgn⁡(wx+b)=sgn⁡(∑i=1nyiαi〈xi·x〉+b),

where **w** is weight vector of the classifier. The optimized weight vector equals to ∑_*i*=1_^*n*^ *α*_*i*_ *y*_*i*_x_*i*_, where *α*_*i*_ is nonzero if *i* belongs to support vector, otherwise *α*_*i*_ equals to 0. Hence, for support vector machine, the binary decision function is determined by the linear combination of support vectors. The class of new sample x can be easily determined by the sign of *f*(*x*). For achieving the objective of better feature selection, Guyon et al. [16] proposed SVMRFE method to find important gene subset for cancer classification. To evaluate the importance of features, SVMRFE uses *w*_*i*_^2^ as ranking criterion. Features with the smallest ranking scores are eliminated. This criterion can also be interpreted as weighted sums of support vectors [17]. In addition, based on SVMRFE, Duan et al. [18] used cross-validation method to estimate multiple *w*_*i*_^2^ and take signal-to-noise ratio of the multiple weight value as the ranking criterion. Hence, the multiple weights SVMRFE is robust to small perturbations for single experiment.

### 2.2. RSVM

To enhance the robustness to noise and outliers, Zhang et al. [17] proposed recursive support vector machine (i.e., RSVM). They developed a feature selection method to construct the stepwise classification rules and reduce the gene numbers at the same time. From (2), one can obtain the weight of specific feature. Unlike SVMRFE, RSVM selects important features by the value of the product of weight and corresponding feature:

(3) wj(mj+−mj−),

where *w*_*j*_ denotes weight of the *j*th feature, *m*_*j*_^+^ and *m*_*j*_^−^ stand for the means of feature *j* in the respective classes. This method takes both the classifier information (i.e., weight) and the data (i.e., class mean of two classes) into consideration.

With the ranking score calculated from (3), we can set a threshold to filter out any features with score below it in the next steps. Furthermore, the classification can be performed with the selected features step by step. After a series of iterations with smaller and smaller feature sets, the best rule is constructed by a prespecified number of features selected from the highest selected-frequency list. Finally, the best rule and number of selected features can be decided simultaneously. However, such feature selection result is greatly affected by class label assignment. We found that different assignments of class labelling (+ or −) will result in different selected gene sets. Hence, such unstable criterion is ambiguous to use.

### 2.3. Proposed SVM-*t* Method

Support vector machine uses only the information of support vectors to construct the maximal separation hyperplane and determine the classes for new samples. The support vectors, the set of closest points between two classes, play an important role in SVMRFE and RSVM for feature selection. These two methods use the weights of corresponding features to build the selection criteria; instead, we combined the univariate ranking methods (i.e., absolute *t*-statistics) with support vector machine. The proposed method uses the most important subset (i.e., SVs) of the data points to construct the selection criteria (4). In other words, we use the algorithm of support vector machine as a sampling technique for data points of two classes simultaneously. The standard two-sample *t*-statistic is used as a surrogate statistic to evaluate the significant differences between two classes. Our proposed method is easy to implement and keeping the computational complexity comparable to that of SVMRFE and RSVM. Therefore, with the variation of samples, we can identify the most significant differences for specific genes among the closest points:

(4) |tj|=|(uj+−uj−)((sj+)2/n+)+((sj−)2/n−)|,

where *n* ^+^ (resp., *n* ^−^) indicates the number of support vectors for class +1 (resp., −1). We can calculate mean *u*_*j*_^+^ (resp., *u*_*j*_^−^) and standard deviation *s*_*j*_^+^ (resp., *s*_*j*_^−^) by using only the support vectors of feature *j*-labelled class +1 (resp., −1) to obtain the score of each feature. The features with highest scores are the features with most significant difference between the two classes. It is intuitive to select features with the highest score as our feature set.

However, there are some exceptions according to such a criterion. We further break down the selection criterion into four situations as follows:

(a) the data set can be well separated by two SVs. Both classes have only one SV. Equation (4) can thus be reduced as the difference of particular feature of SVs;
(b) one class has only one SV, and the other class has more than one SV, and (4) becomes a single mean *t*-test statistics. Considering class −1 with only one SV, (4) reduces to
  (5) |tj|=|(uj+−uj−)(sj+)2/n+|;
(c) similar to (b), class +1 has only one SV. The selection criterion is
  (6) |tj|=|(uj+−uj−)(sj−)2/n−|;
(d) finally, in the most general condition, we usually encounter classification problems with more than one support vectors for each class. For practical use, this condition is more complicated than previous ones, and selection criterion in (4) shall be used.

**Figure 1:**
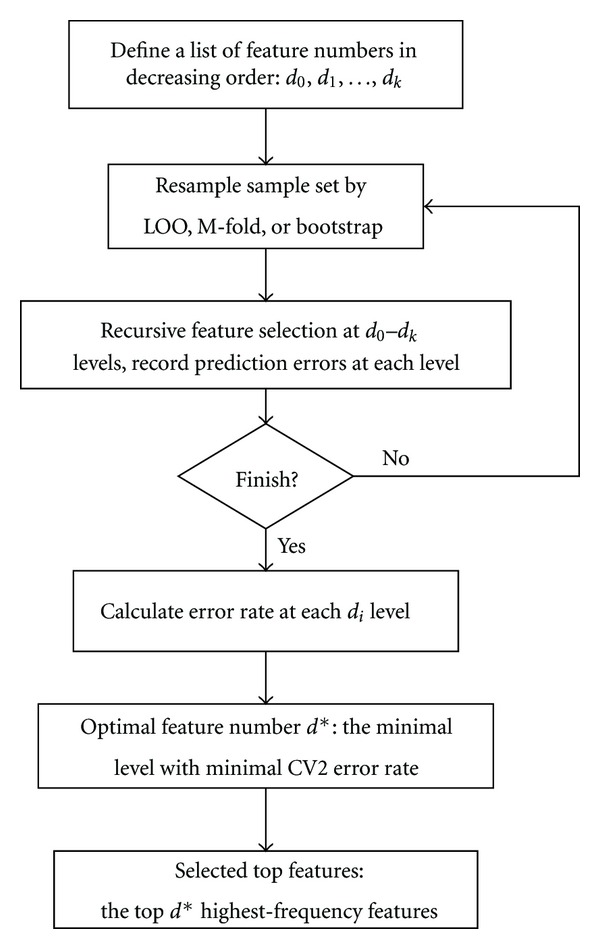
Workflow of feature selection. Workflow of the SVM-*t* algorithm.

We follow the workflow of feature selection suggested in [17] as in Figure 1. First, a monotone decreasing sequence {*d*_*k*_,…, *d*_0_} for the number of selected features is specified where *k* is the total number of runs. After executing the selection criteria for each run, the number of input features *d*_*k*_ will be determined. Secondly, the cross-validation method is adopted to carry out gene selection procedure and execute the SVM procedures with *d*_*i*_ features. For the comparison among the difference of selection methods, we apply these three different criteria in this step. For each run, the features with top *d*_*i*+1_ ranking scores are selected until the sequence ends. Finally, the gene set with minimal CV error is selected. The set with fewest genes will be chosen when ties occur for CV errors.

## 3. Results

### 3.1. Simulation Experiments

First, we evaluate performance of the three methods using simulated data sets. Our simulation data contains different means and standard deviations for informative and noninformative genes. We first generate a training set with 100 samples (50 samples for each class), each containing expression values of 1000 genes where 300 of them are informative genes and the rest 700 noninformative genes. To validate the selected gene sets, we also generate another 1000 independent samples (500 samples for each class). We performed 100 simulations for each data set and used “Leave-one-out” version CV method. The following two simulation cases use the same aforementioned scheme with varying parameter setting.

**Simulation Case I**

In this simulation, we separate the informative gene set into two parts. The first 150 genes independently follow the Gaussian distribution *N*(0.15, 0.5) for class 1 and *N*(−0.15, 0.5) for class 2. On the contrary, the last 150 genes are independently distributed from the Gaussian distribution *N*(−0.15, 0.5) for class 1 and *N*(0.15, 0.5) for class 2. The rest 700 “noninformative” genes independently follow *N*(0,1) distribution.  Table 1 summarizes the simulation result with 100 runs using average and standard deviation of the 100 runs with the percentage of informative genes coverage and the number of support vectors (SVs). With this parameter setting, the informative genes cannot be easily differentiated due to the small mean difference. However, taking the standard deviation into consideration, we found that the proposed method shows better results than RSVM and SVMRFE.

In addition, we increase the standard deviation for informative genes to 1 so that the difference of variation effect between informative and noninformative genes is reduced.  Table 2 shows that RSVM yields better average test error rate and selects more informative genes than SVMRFE and the proposed method. However, the proposed method selected fewer support vectors in each recursive step.

Based on the previous setting, we further increase the variance of noninformative genes. We set the standard deviation of noninformative gene twice as large as that of informative genes and follows *N*(0,2) distribution.  Table 3 shows that all three methods give worse test error rates than before because of the more complex nature of the simulation scheme. The coverage percentages of informative genes by RSVM and SVMRFE decrease with the decreasing gene levels, but the proposed method maintains high coverage percentages of informative genes. Besides, the test error rate of proposed method stays at around 25% which is lower than the error rates of RSVM and SVMRFE.

**Simulation Case II**

Here, we increase the mean values of the informative genes from 0.15 to 0.25 and keep the distribution of the 700 “noninformative” at *N*(0,1). In Table 4, we find that this simulated data is well separated with the higher difference between the two classes. The test error rate and percentage of selected informative genes are comparable among the three methods. The prediction results are almost perfect (i.e., test errors are nearly zero) for all conditions in this simulation. The proposed method utilizes fewer support vectors than the other two methods though.

We further set standard deviation of noninformative to 1 to eliminate the variation effect between informative and noninformative genes. The result in Table 5 is very similar to that of Table 2 with lower test error rates for the three methods. With decreasing gene number, RSVM gives better average test error rate than SVMRFE and proposed method. The percent of selected informative genes are comparable among the three methods which may due to the higher difference of informative genes between two classes. In addition, our proposed method also selected fewer support vectors.

Finally, we set the standard deviation of noninformative gene twice as large as that of informative genes with distribution *N*(0,2). The three methods all yield higher test error rate than previous settings but the proposed method performs better than RSVM and SVMRFE with significant margins for such complex simulation data.

**Stability of Feature Selection**

**Figure 2:**
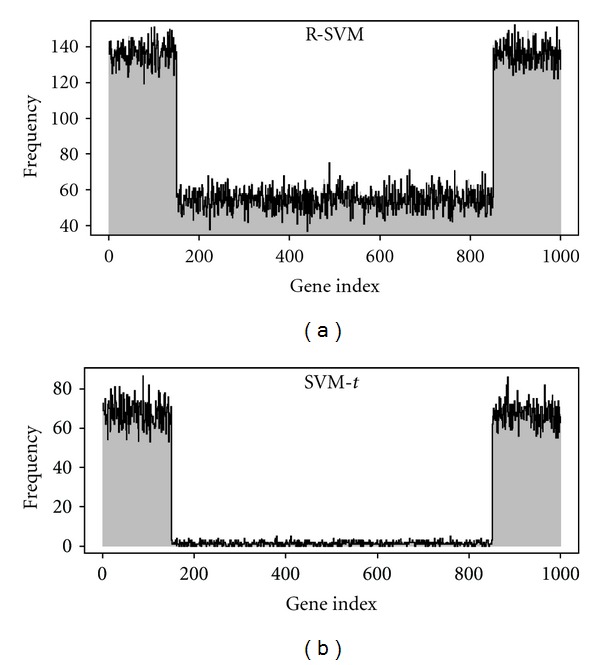
Stability of feature selection for switching class labels. Grey vertical and solid black lines represent frequencies of selected features for different class labels. The first 150 genes and the last 150 genes are discriminative genes.

**Figure 3:**
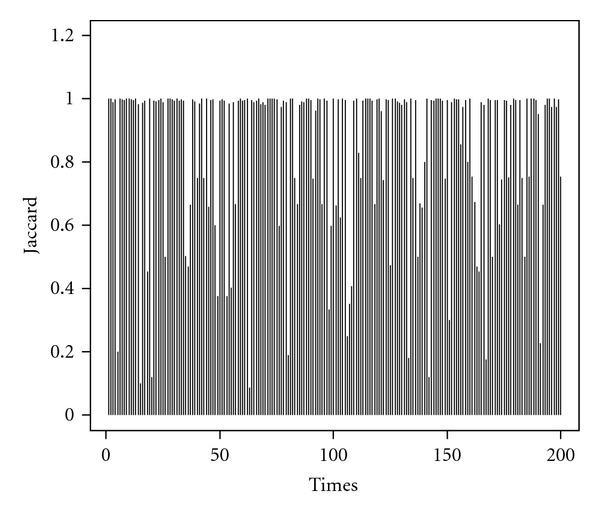
Jaccard's coefficients over 200 simulations for RSVM.

To investigate the stability of feature selection, we perform 200 times of simulation case I with switched class labels. The frequency plot of selected features in Figure 2 shows that different class labels in RSVM will result in selecting the different sets of features, while our proposed method does not alter the frequencies of selected features by switching the class labels. In addition, the RSVM tends to select noninformative genes much more frequently than our approach.  Figure 3 plots the Jaccard's coefficient of RSVM to comparing the similarity of feature selections between two switched class labels over 200 simulations. We observe that the class labelling in RSVM is crucial for achieving better agreement of feature selection. The low Jaccard's coefficient reveals that there is heterogeneity between two different class labels for some simulations. In contrast to the RSVM, our proposed method is independent of the class labels and appears to be better than RSVM for selecting a small number of discriminative genes.

### 3.2. Application on the Human Breast Cancer Data

We next evaluated performance of the three methods by using two microarray datasets. The first dataset is from Affymetrix DNA microarray analysis of a series of primary breast cancer samples [9]. This data contains gene expression profiles of 7129 detected probe sets for 49 samples (24 ER positive and 25 negative patients). Preprocessings including background correction, normalization, PM correction and expression summary were conducted, and the gene expression levels were log2-transformed. All 7129 genes were included in the gene selection process. The minimal gene number is set at 5 with decreasing ration 0.85 for generating the decreasing gene selection sequence (Table 6).

Table 7 shows that RSVM selects fewer discriminant genes than SVMRFE and the proposed method. However, in earlier recursive steps, the proposed method attains the minimal CV error rate (2.04%). Proposed method may select more significant genes than SVMRFE and RSVM in the first few iterations. Taking also gene number into consideration we may choose 10 genes with CV error rate (8.16%) as our solution. SVMRFE can also attain CV error rate (8.16%) with 20 selected genes and RSVM can attain minimal error rate (4.08%) with 5 genes. For gene number fewer than 20, proposed method has higher number of support vectors than SVMRFE and RSVM, and these two methods have almost identical number of support vectors.  Table 8 shows that proposed method selected 10 genes with *P* value < 0.0004.

### 3.3. Application on the Lung Cancer Data

The second dataset is a lung cancer dataset used in CAMDA 2003 [19]. Gene expression profiles of 7129 genes for 86 lung cancer patients (67 stage I and 19 stage III tumors) are available for analysis. Similar preprocessing and parameter setting to the breast cancer analysis are adopted.

 Error rates shown in Table 9 tell us that this lung cancer data is more difficult than the breast cancer data for predicting the disease subtypes. SVM-RFE and RSVM can only reach error rate at above 20%, while the proposed method attains CV error rate below 10% at several feature levels.  Table 10 shows the selected genes by the proposed method, and most of them have *P* values less than 0.05.

## 4. Discussion

The proposed SVM-*t* method is a multivariate feature selection approach in which sample heterogeneity is accommodated during a sequential backward elimination process. The method could benefit from the use of support vectors on the hyperplane of SVM and select a group of informative genes with aids in improving classification performance. The use of support vectors for feature selection is intuitive because they play an important role for building the decision function in SVM. For the linear SVM, the feature dimensions corresponding to the support vectors are also critical for classification by the property of linearity. Therefore, we consider trimmed *t*-statistics which compare group mean values after removing the nonsupport vectors and show that these have higher power to identify relevant features than the other two SVM-based approaches in case of high noise.

The result for simulation studies show that all three feature selection methods of SVMRFE, RSVM, and the proposed method have good performance when the distribution of differentially expressed genes is much different from noise genes. The influences could be attributed to two sources: the magnitude of difference and variance of gene expression levels between two classes. When fixing the magnitude of difference, the result is greatly affected by the variance. With identical variance of informative and noninformative genes, RSVM performs better result since it gives more weights to informative genes (i.e., genes with higher difference between the two classes become more important in this case). On the other hand, when considering distinct variances of informative and noninformative genes, the proposed method outperforms RSVM and SVMRFE. Moreover, the proposed method is able not only to select more informative genes with lower test error rate than RSVM and SVMRFE but also to accommodate heterogeneity within the complex clinical samples.

In applications, we use two published microarray datasets to evaluate the performance of the three methods via leave-one-out cross-validated accuracy. We can find that proposed method can consistently select a smaller subset of informative genes with good prediction accuracy. Both the proposed method and RSVM outperform SVMRFE for applications on the two datasets.

All SVM-based feature selection methods under comparisons select features based on the support vectors in the linear SVM. Ideally, SVMs can use a proper kernel function to map the data into a separating hyperplane in the feature space when the data points are not linearly separable. However, the corresponding property between support vectors and feature dimensions in the nonlinear SVM is far from being clear and some further work is required in order to fully understand this issue. In addition, it is well known that applying proper kernel functions and parameters to a specific real database remains challenging, and the number of support vectors that compose the decision function increases dramatically when the decision manifold becomes complicated. Therefore, our approach is limited to the use of linear SVM here and considers it as an alternative approximation to the real dataset. However, if the objective is to select relevant features for better classification performance, rather than to provide insights into relative importance of features in the feature space, then our approach provides an alternative solution. Further work will be focused on the impact of our trimmed *t*-statistics on the use of nonlinear SVMs for classification of nonlinear datasets.

## Figures and Tables

**Table 1:** Comparison of selection methods on simulation data 1.

Num. of genes	RSVM			SVMRFE			SVM-*t*		
	Test (%)	Rec (%)	nSV	Test (%)	Rec (%)	nSV	Test (%)	Rec (%)	nSV
800	1.06 ± 0.37	98.97 ± 0.51	96.42 ± 0.08	1.07 ± 0.37	98.37 ± 0.63	98.25 ± 0.04	0.97 ± 0.37	99.59 ± 0.37	95.36 ± 0.07
600	0.94 ± 0.36	93.76 ± 1.26	92.90 ± 0.12	0.99 ± 0.39	92.49 ± 1.44	97.26 ± 0.09	0.67 ± 0.29	98.52 ± 0.61	87.45 ± 0.45
500	0.92 ± 0.38	87.38 ± 1.70	90.62 ± 0.16	0.95 ± 0.38	85.95 ± 1.91	95.96 ± 0.13	0.44 ± 0.24	96.96 ± 0.87	81.14 ± 1.09
400	1.02 ± 0.42	77.00 ± 2.05	84.91 ± 0.21	1.01 ± 0.38	76.34 ± 2.20	90.52 ± 0.20	0.19 ± 0.15	93.48 ± 1.34	71.48 ± 1.06
300	1.26 ± 0.54	63.12 ± 2.06	76.51 ± 0.29	1.31 ± 0.60	62.99 ± 2.09	80.02 ± 0.30	0.04 ± 0.07	84.28 ± 1.47	60.63 ± 0.91
200	2.14 ± 0.92	67.68 ± 2.89	61.99 ± 0.37	2.07 ± 0.93	68.56 ± 2.83	63.93 ± 0.40	0.04 ± 0.6	94.89 ± 1.78	49.37 ± 0.99
150	2.88 ± 1.13	71.35 ± 3.83	56.73 ± 0.40	2.61 ± 1.25	72.76 ± 3.70	59.05 ± 0.37	0.10 ± 0.12	97.79 ± 1.49	43.67 ± 1.21
100	4.36 ± 1.93	74.62 ± 4.75	43.35 ± 0.32	3.75 ± 1.76	77.62 ± 4.61	44.48 ± 0.28	0.53 ± 0.28	99.14 ± 1.05	36.07 ± 0.75
90	4.58 ± 2.02	76.32 ± 4.73	45.30 ± 0.30	4.01 ± 1.91	79.34 ± 4.77	46.57 ± 0.31	0.79 ± 0.34	99.14 ± 1.00	34.64 ± 0.82
80	5.20 ± 2.21	77.29 ± 4.85	42.66 ± 0.31	4.21 ± 1.86	81.33 ± 4.80	43.66 ± 0.32	1.14 ± 0.44	99.41 ± 1.01	32.81 ± 0.69
70	5.64 ± 2.61	78.84 ± 5.28	39.27 ± 0.33	4.47 ± 1.90	83.10 ± 5.12	40.04 ± 0.27	1.66 ± 0.58	99.60 ± 0.86	30.81 ± 0.54
60	6.41 ± 2.56	80.37 ± 5.39	35.21 ± 0.26	5.02 ± 1.97	85.62 ± 5.14	35.82 ± 0.32	2.57 ± 0.72	99.65 ± 1.04	28.62 ± 0.36
50	7.43 ± 2.57	82.44 ± 5.85	30.89 ± 0.27	5.77 ± 2.09	88.06 ± 5.67	31.21 ± 0.25	3.91 ± 0.89	99.62 ± 0.93	26.32 ± 0.41

Num. of genes: number of selected features in each recursive step. Results of 1000 features are the same and we do not list them here.

Test: test error rate.

Rec: the percentage of recovery informative genes (among 300 informative genes).

nSV: the average number of support vectors selected in each recursive step.

**Table 2:** Comparison of selection methods on simulation data 2.

Num. of genes	RSVM			SVMRFE			SVM-*t*		
	Test (%)	Rec (%)	nSV	Test (%)	Rec (%)	nSV	Test (%)	Rec (%)	nSV
800	6.15 ± 0.84	91.24 ± 1.55	96.60 ± 0.09	6.06 ± 0.85	90.48 ± 1.40	98.01 ± 0.06	6.24 ± 0.82	91.59 ± 1.32	96.02 ± 0.14
600	6.44 ± 0.88	80.45 ± 2.26	94.58 ± 0.14	6.28 ± 0.86	78.86 ± 2.27	97.60 ± 0.09	6.72 ± 0.88	81.19 ± 1.97	91.29 ± 0.41
500	6.64 ± 0.93	73.63 ± 2.39	92.98 ± 0.15	6.51 ± 0.91	71.95 ± 2.26	97.15 ± 0.10	7.15 ± 0.97	74.24 ± 2.14	87.72 ± 0.99
400	7.23 ± 1.04	65.58 ± 2.39	89.59 ± 0.25	7.11 ± 1.03	63.74 ± 2.45	95.19 ± 0.21	7.91 ± 1.23	65.67 ± 2.48	81.56 ± 1.58
300	8.07 ± 1.01	55.53 ± 2.18	83.60 ± 0.36	8.05 ± 1.19	53.62 ± 2.23	88.72 ± 0.30	9.13 ± 1.50	55.35 ± 2.40	73.08 ± 1.79
200	10.05 ± 1.48	63.29 ± 3.17	71.21 ± 0.51	10.32 ± 1.51	61.08 ± 3.11	74.08 ± 0.51	11.90 ± 1.59	62.46 ± 2.85	61.41 ± 1.52
150	11.80 ± 1.82	67.97 ± 3.52	65.14 ± 0.45	12.16 ± 1.83	65.53 ± 3.87	69.57 ± 0.45	14.16 ± 1.68	67.13 ± 3.29	54.77 ± 1.21
100	14.81 ± 1.92	74.24 ± 4.15	50.51 ± 0.40	15.37 ± 1.94	71.13 ± 4.12	52.82 ± 0.49	18.09 ± 2.04	72.74 ± 3.56	45.75 ± 0.50
90	15.70 ± 1.76	75.37 ± 4.17	52.23 ± 0.40	16.24 ± 1.93	72.36 ± 4.58	54.89 ± 0.43	19.28 ± 2.37	74.02 ± 3.98	43.81 ± 0.40
80	16.70 ± 1.87	76.80 ± 4.14	49.25 ± 0.46	17.34 ± 1.88	74.05 ± 4.72	51.68 ± 0.40	20.51 ± 2.26	75.19 ± 4.47	41.50 ± 0.39
70	17.94 ± 1.86	78.27 ± 4.40	45.24 ± 0.43	18.61 ± 1.85	75.73 ± 4.34	47.22 ± 0.38	22.13 ± 2.35	76.69 ± 4.50	39.12 ± 0.47
60	19.28 ± 1.87	80.15 ± 4.85	40.49 ± 0.41	19.91 ± 2.12	77.18 ± 4.83	42.17 ± 0.41	23.91 ± 2.36	77.63 ± 5.03	36.59 ± 0.66
50	21.21 ± 1.87	82.22 ± 4.79	35.40 ± 0.37	21.55 ± 1.94	79.06 ± 4.94	36.58 ± 0.36	25.83 ± 2.66	79.90 ± 5.59	33.86 ± 0.64

Num. of genes, Test, Rec, and nSV: the same as Table 1.

**Table 3:** Comparison of selection methods on simulation data 3.

Num. of genes	RSVM			SVMRFE			SVM-*t*		
	Test (%)	Rec (%)	nSV	Test (%)	Rec (%)	nSV	Test (%)	Rec (%)	nSV
800	26.39 ± 1.65	86.52 ± 1.60	97.68 ± 0.04	26.66 ± 1.76	84.41 ± 1.75	98.63 ± 0.03	26.13 ± 1.65	91.56 ± 1.38	97.07 ± 0.05
600	27.36 ± 1.64	67.53 ± 2.06	96.16 ± 0.1	27.99 ± 1.70	65.72 ± 2.26	98.41 ± 0.04	25.46 ± 1.74	81.20 ± 2.09	91.60 ± 0.29
500	28.63 ± 1.72	56.83 ± 2.18	95.08 ± 0.11	29.12 ± 1.67	55.57 ± 2.27	98.17 ± 0.05	25.05 ± 1.70	74.70 ± 2.08	87.77 ± 1.05
400	30.42 ± 1.91	44.77 ± 2.40	92.24 ± 0.16	30.76 ± 1.95	44.57 ± 2.34	96.53 ± 0.10	24.31 ± 2.17	66.70 ± 2.15	80.84 ± 1.18
300	32.98 ± 1.98	32.58 ± 2.30	87.17 ± 0.20	33.05 ± 2.21	32.90 ± 2.41	90.65 ± 0.25	23.30 ± 2.29	56.71 ± 2.19	71.85 ± 1.17
200	36.59 ± 2.21	30.20 ± 3.00	74.61 ± 0.30	36.49 ± 2.08	31.66 ± 2.84	76.09 ± 0.31	22.26 ± 2.53	65.50 ± 2.95	60.63 ± 0.66
150	38.31 ± 2.04	29.55 ± 3.65	68.60 ± 0.33	38.09 ± 2.22	31.37 ± 3.61	71.66 ± 0.35	21.60 ± 2.93	71.07 ± 3.79	54.36 ± 0.48
100	40.85 ± 2.20	27.15 ± 4.50	53.31 ± 0.33	40.37 ± 2.45	30.40 ± 4.40	54.78 ± 0.33	21.53 ± 3.19	77.63 ± 4.91	46.03 ± 0.49
90	41.27 ± 2.19	27.43 ± 4.76	54.61 ± 0.30	40.63 ± 2.52	30.49 ± 5.09	56.22 ± 0.30	21.90 ± 3.37	78.89 ± 5.06	44.07 ± 0.54
80	41.62 ± 1.96	27.61 ± 4.88	51.27 ± 0.30	40.61 ± 2.39	31.05 ± 5.44	52.55 ± 0.30	22.44 ± 3.51	80.35 ± 5.32	41.95 ± 0.56
70	41.99 ± 2.40	27.56 ± 5.29	47.08 ± 0.28	41.17 ± 2.61	31.47 ± 5.84	48.03 ± 0.32	22.90 ± 3.41	81.69 ± 5.40	39.66 ± 0.64
60	42.51 ± 2.16	26.98 ± 5.34	42.10 ± 0.27	41.56 ± 2.56	31.90 ± 6.17	42.73 ± 0.27	24.18 ± 3.12	83.02 ± 5.41	36.98 ± 0.63
50	42.99 ± 2.56	26.84 ± 6.27	36.77 ± 0.27	41.53 ± 2.71	32.52 ± 6.59	37.05 ± 0.27	25.56 ± 3.30	84.58 ± 6.10	34.18 ± 0.66

Num. of genes, Test, Rec, and nSV: the same as Table 1.

**Table 4:** Comparison of selection methods on simulation data 4.

Num. of genes	RSVM			SVMRFE			SVM-*t*		
	Test (%)	Rec (%)	nSV	Test (%)	Rec (%)	nSV	Test (%)	Rec (%)	nSV
800	0.00 ± 0.00	100.00	93.82 ± 0.09	0.00 ± 0.00	100.00	96.88 ± 0.06	0.00 ± 0.00	100.00	92.54 ± 0.10
600	0.00 ± 0.00	99.95 ± 0.13	88.22 ± 0.15	0.00 ± 0.00	99.88 ± 0.19	94.37 ± 0.13	0.00 ± 0.00	100.00	82.03 ± 0.77
500	0.00 ± 0.00	99.68 ± 0.32	82.72 ± 0.20	0.00 ± 0.00	99.39 ± 0.45	90.14 ± 0.17	0.00 ± 0.00	99.99 ± 0.06	73.90 ± 1.23
400	0.00 ± 0.00	98.02 ± 0.77	70.36 ± 0.24	0.00 ± 0.00	96.80 ± 1.01	77.60 ± 0.28	0.00 ± 0.00	99.94 ± 0.14	61.64 ± 0.83
300	0.00 ± 0.00	87.36 ± 1.37	57.45 ± 0.32	0.00 ± 0.00	85.18 ± 1.43	63.84 ± 0.39	0.00 ± 0.00	97.26 ± 0.76	46.16 ± 0.33
200	0.00 ± 0.00	94.49 ± 1.63	46.07 ± 0.31	0.00 ± 0.00	93.15 ± 1.58	48.56 ± 0.32	0.00 ± 0.00	99.94 ± 0.18	37.89 ± 1.60
150	0.00 ± 0.00	96.91 ± 1.61	42.99 ± 0.33	0.00 ± 0.00	96.60 ± 1.80	45.50 ± 0.35	0.00 ± 0.00	99.96 ± 0.16	33.85 ± 2.04
100	0.00 ± 0.00	98.66 ± 1.28	33.32 ± 0.29	0.00 ± 0.00	98.72 ± 1.36	34.71 ± 0.32	0.00 ± 0.00	99.89 ± 0.35	27.87 ± 1.13
90	0.00 ± 0.00	99.03 ± 1.09	36.60 ± 0.31	0.00 ± 0.00	98.92 ± 1.31	38.08 ± 0.35	0.00 ± 0.00	99.88 ± 0.44	26.93 ± 1.30
80	0.00 ± 0.00	99.33 ± 1.07	34.94 ± 0.28	0.00 ± 0.00	99.35 ± 0.98	36.35 ± 0.29	0.00 ± 0.00	99.90 ± 0.34	25.55 ± 1.13
70	0.01 ± 0.02	99.43 ± 0.97	32.49 ± 0.29	0.01 ± 0.02	99.60 ± 0.86	33.62 ± 0.33	0.01 ± 0.03	99.90 ± 0.42	23.97 ± 0.95
60	0.01 ± 0.04	99.55 ± 0.88	29.40 ± 0.27	0.02 ± 0.04	99.53 ± 0.92	30.31 ± 0.32	0.03 ± 0.06	99.88 ± 0.43	22.30 ± 0.67
50	0.03 ± 0.07	99.76 ± 0.71	25.91 ± 0.26	0.04 ± 0.07	99.78 ± 0.69	26.65 ± 0.29	0.09 ± 0.1	99.84 ± 0.55	20.47 ± 0.48

Num. of genes, Test, Rec, and nSV: the same as Table 1.

**Table 5:** Comparison of selection methods on simulation data 5.

Num. of genes	RSVM			SVMRFE			SVM-*t*		
	Test (%)	Rec (%)	nSV	Test (%)	Rec (%)	nSV	Test (%)	Rec (%)	nSV
800	0.05 ± 0.07	98.09 ± 0.78	92.07 ± 0.08	0.05 ± 0.07	97.26 ± 0.90	95.12 ± 0.07	0.05 ± 0.07	98.44 ± 0.70	91.05 ± 0.11
600	0.06 ± 0.08	94.89 ± 1.14	88.21 ± 0.13	0.04 ± 0.06	93.00 ± 1.37	93.74 ± 0.10	0.05 ± 0.07	95.35 ± 1.09	83.85 ± 0.72
500	0.06 ± 0.08	91.47 ± 1.39	85.42 ± 0.15	0.05 ± 0.07	88.91 ± 1.76	92.29 ± 0.12	0.06 ± 0.08	92.08 ± 1.58	79.30 ± 1.23
400	0.06 ± 0.08	86.07 ± 1.81	80.50 ± 0.17	0.06 ± 0.08	82.49 ± 2.00	88.29 ± 0.17	0.08 ± 0.09	85.86 ± 1.90	72.49 ± 1.66
300	0.07 ± 0.08	75.82 ± 2.03	73.51 ± 0.24	0.08 ± 0.10	71.78 ± 2.15	80.72 ± 0.28	0.11 ± 0.11	75.15 ± 2.00	63.95 ± 1.81
200	0.17 ± 0.13	87.61 ± 2.00	61.22 ± 0.29	0.21 ± 0.16	82.71 ± 2.30	65.46 ± 0.33	0.30 ± 0.24	85.78 ± 2.35	53.12 ± 1.72
150	0.34 ± 0.21	92.33 ± 2.23	56.24 ± 0.30	0.40 ± 0.20	88.59 ± 2.51	60.89 ± 0.31	0.69 ± 0.37	90.68 ± 2.67	47.09 ± 1.60
100	1.17 ± 0.44	96.48 ± 1.99	43.75 ± 0.32	1.28 ± 0.38	93.68 ± 2.59	46.26 ± 0.35	2.15 ± 0.65	94.29 ± 2.63	38.94 ± 0.68
90	1.45 ± 0.47	97.01 ± 2.11	46.10 ± 0.26	1.63 ± 0.49	94.88 ± 2.49	48.90 ± 0.34	2.74 ± 0.74	95.09 ± 2.71	37.32 ± 0.63
80	1.90 ± 0.59	97.83 ± 1.97	43.71 ± 0.32	2.08 ± 0.56	95.70 ± 2.49	46.21 ± 0.31	3.52 ± 0.88	95.88 ± 2.92	35.30 ± 0.45
70	2.51 ± 0.65	98.26 ± 1.88	40.37 ± 0.30	2.79 ± 0.67	96.49 ± 2.66	42.40 ± 0.32	4.60 ± 1.06	96.74 ± 2.50	33.16 ± 0.32
60	3.52 ± 0.71	98.82 ± 1.54	36.36 ± 0.31	3.72 ± 0.82	97.33 ± 2.48	37.93 ± 0.30	5.92 ± 1.25	97.52 ± 2.59	30.80 ± 0.39
50	4.94 ± 0.87	99.10 ± 1.46	31.99 ± 0.26	5.00 ± 0.89	98.32 ± 2.00	33.11 ± 0.29	7.93 ± 1.43	97.88 ± 2.46	28.32 ± 0.45

Num. of genes, Test, Rec, and nSV: the same as Table 1.

**Table 6:** Comparison of selection methods on simulation data 6.

Num. of genes	RSVM			SVMRFE			SVM-*t*		
	Test (%)	Rec (%)	nSV	Test (%)	Rec (%)	nSV	Test (%)	Rec (%)	nSV
800	4.96 ± 0.90	96.92 ± 0.90	96.88 ± 0.07	5.02 ± 0.91	95.78 ± 1.07	98.43 ± 0.04	4.72 ± 0.86	98.63 ± 0.64	95.99 ± 0.07
600	4.91 ± 0.96	87.79 ± 1.62	94.16 ± 0.11	5.09 ± 1.03	86.10 ± 1.66	97.77 ± 0.07	3.86 ± 0.83	95.76 ± 1.11	88.66 ± 0.44
500	5.30 ± 1.05	79.05 ± 1.97	92.60 ± 0.13	5.46 ± 1.15	77.77 ± 2.11	97.07 ± 0.11	3.06 ± 0.75	92.73 ± 1.29	83.23 ± 1.19
400	5.88 ± 1.28	67.74 ± 2.33	88.34 ± 0.21	6.10 ± 1.31	67.05 ± 2.56	93.20 ± 0.20	2.09 ± 0.72	87.26 ± 1.66	74.71 ± 1.06
300	7.34 ± 1.64	64.62 ± 2.63	81.00 ± 0.29	7.44 ± 1.62	64.78 ± 2.97	83.88 ± 0.27	1.22 ± 0.53	92.60 ± 2.10	64.77 ± 1.00
200	9.99 ± 1.98	56.40 ± 2.97	66.42 ± 0.36	9.85 ± 2.05	57.43 ± 2.98	68.16 ± 0.35	0.85 ± 0.46	88.83 ± 1.94	53.17 ± 0.80
150	11.91 ± 2.41	58.77 ± 3.58	60.70 ± 0.37	11.40 ± 2.24	60.39 ± 3.62	63.23 ± 0.38	1.00 ± 0.51	93.74 ± 2.25	46.96 ± 0.83
100	14.84 ± 2.91	60.30 ± 4.22	46.77 ± 0.30	13.49 ± 2.82	64.10 ± 4.28	47.76 ± 0.35	2.19 ± 0.78	96.98 ± 2.36	39.08 ± 0.48
90	15.62 ± 3.17	61.60 ± 4.68	48.39 ± 0.29	13.62 ± 2.90	65.53 ± 4.86	49.56 ± 0.30	2.67 ± 0.95	97.36 ± 2.11	37.39 ± 0.58
80	15.85 ± 3.37	63.10 ± 5.30	45.47 ± 0.33	14.23 ± 3.15	67.33 ± 5.37	46.45 ± 0.33	3.43 ± 0.90	97.93 ± 2.02	35.39 ± 0.41
70	16.70 ± 3.62	64.47 ± 5.78	41.75 ± 0.31	14.67 ± 3.46	69.53 ± 5.70	42.48 ± 0.31	4.30 ± 1.03	98.19 ± 1.90	33.25 ± 0.36
60	17.56 ± 3.88	65.48 ± 6.38	37.47 ± 0.29	15.25 ± 3.55	71.52 ± 5.99	37.87 ± 0.28	5.69 ± 1.29	98.65 ± 1.75	30.95 ± 0.38
50	18.80 ± 4.01	66.70 ± 6.91	32.81 ± 0.28	15.78 ± 3.54	74.04 ± 6.63	33.03 ± 0.29	7.42 ± 1.32	99.10 ± 1.67	28.44 ± 0.41

Num. of genes, Test, Rec, and nSV: the same as Table 1.

**Table 7:** The CV results on the breast cancer dataset.

Num. of genes	RSVM		SVM-RFE		SVM-*t*	
	CV2	Mean SV	CV2	Mean SV	CV2	Mean SV
7129	8.16%	47.02	8.16%	47.02	8.16%	47.02
6060	8.16%	45.94	8.16%	47.02	6.12%	46.88
5151	8.16%	45.31	8.16%	47.02	6.12%	44.88
4378	8.16%	44.55	8.16%	47.02	6.12%	42.84
3721	8.16%	43.20	8.16%	47.00	6.12%	39.55
3163	8.16%	41.98	8.16%	47.00	4.08%	37.41
2689	8.16%	40.92	8.16%	46.84	2.04%	35.67
2286	10.20%	39.63	10.20%	46.78	2.04%	35.18
1943	10.20%	38.41	12.24%	46.61	4.08%	34.31
1652	10.20%	37.47	12.24%	46.06	4.08%	32.43
1404	10.20%	36.41	12.24%	45.39	4.08%	32.31
1193	12.24%	35.31	12.24%	44.80	6.12%	30.90
1014	12.24%	34.12	12.24%	44.39	6.12%	30.24
862	12.24%	33.04	12.24%	43.14	6.12%	29.76
733	12.24%	32.10	12.24%	42.27	4.08%	29.02
623	12.24%	30.84	10.20%	41.31	6.12%	27.10
530	12.24%	30.10	12.24%	40.96	6.12%	26.16
450	12.24%	29.29	12.24%	39.49	6.12%	24.35
382	12.24%	29.00	10.20%	38.04	8.16%	23.37
325	10.20%	28.78	12.24%	37.49	8.16%	22.33
276	10.20%	28.14	10.20%	36.59	6.12%	20.78
235	12.24%	27.55	12.24%	35.27	6.12%	19.94
200	10.20%	26.67	12.24%	34.14	8.16%	19.24
170	10.20%	25.98	10.20%	33.53	8.16%	18.14
144	14.29%	25.12	12.24%	31.67	10.20%	17.94
122	14.29%	23.84	12.24%	30.67	12.24%	17.63
104	12.24%	22.12	12.24%	29.65	12.24%	17.53
88	10.20%	21.02	14.29%	27.67	14.29%	16.76
75	10.20%	20.39	14.29%	25.92	12.24%	16.78
64	12.24%	19.39	8.16%	24.61	14.29%	16.43
54	12.24%	18.37	8.16%	22.14	18.37%	16.33
46	10.20%	18.10	8.16%	21.57	20.41%	15.57
39	14.29%	16.61	10.20%	19.24	16.33%	15.57
33	14.29%	15.55	10.20%	16.86	16.33%	15.04
28	16.33%	13.98	10.20%	15.96	16.33%	14.31
24	16.33%	13.04	10.20%	15.02	18.37%	13.69
20	12.24%	12.02	8.16%	12.78	22.45%	13.39
17	10.20%	11.20	14.29%	11.65	22.45%	12.76
14	8.16%	10.29	12.24%	10.59	12.24%	12.43
12	6.12%	9.16	14.29%	9.73	14.29%	12.04
10	4.08%	8.35	14.29%	8.69	8.16%	12.16
8	6.12%	7.29	16.33%	7.51	10.20%	12.22
7	6.12%	7.22	16.33%	6.86	16.33%	12.86
6	8.16%	6.29	16.33%	6.20	12.24%	13.43
5	4.08%	5.98	18.37%	6.20	12.24%	13.63

Num. of genes: the number of selected genes in the recursive steps. CV2: total cross-validation error rate (CV2 error rate). Mean SV: mean number of support vectors used in the cross-validation at each level.

**Table 8:** Selected biomarkers on the breast cancer dataset.

Probe Id	Rank	Gene title	Gene symbol	*t*-statistics	*P* value
X03635_at	1	Estrogen receptor 1	ESR1	9.509	4.08*E*−09
U79293_at	2	Thrombospondin, type I, domain containing 4	THSD4	8.198	2.21*E*−07
U39840_at	3	Forkhead box A1	FOXA1	7.149	4.78*E*−06
X17059_s_at	5	N-acetyltransferase 1 (arylamine N-acetyltransferase)	NAT1	7.175	4.78*E*−06
L08044_s_at	10	Trefoil factor 3 (intestinal)	TFF3	6.328	4.14*E*−05
X83425_at	12	Basal cell adhesion molecule (Lutheran blood group)	BCAM	6.028	1.08*E*−04
M59815_at	14	Complement component 4A (Rodgers blood group) /// complement Component 4B (Childo blood group)	C4A /// C4B	5.927	1.27*E*−04
X76180_at	17	Sodium channel, nonvoltage-gated 1 alpha	SCNN1A	5.796	1.69*E*−04
X53002_s_at	63	Integrin, beta 5	ITGB5	4.639	2.71*E*−03
U52522_at	70	ADP-ribosylation factor interacting protein 2 (arfaptin 2)	ARFIP2	4.533	3.51*E*−03

Rank: gene-ranking is computed using *P* values.

**Table 9:** The CV results on the lung cancer dataset.

Num. of genes	RSVM		SVM-RFE		SVM-*t*	
	CV2	Mean SV	CV2	Mean SV	CV2	Mean SV
7129	22.09%	51.34	22.09%	51.34	22.09%	51.34
6060	20.93%	46.78	22.09%	51.35	20.93%	50.85
5151	20.93%	45.07	22.09%	51.40	19.77%	50.20
4378	20.93%	44.24	22.09%	51.42	18.60%	49.24
3721	20.93%	43.99	22.09%	51.35	15.12%	48.22
3163	20.93%	43.53	22.09%	51.34	15.12%	47.60
2689	20.93%	43.38	22.09%	51.36	15.12%	47.38
2286	20.93%	43.12	20.93%	51.86	15.12%	46.71
1943	22.09%	43.17	20.93%	52.79	12.79%	46.05
1652	22.09%	43.26	20.93%	52.51	12.79%	45.10
1404	22.09%	43.12	20.93%	51.84	13.95%	43.78
1193	22.09%	42.87	20.93%	52.33	10.47%	42.60
1014	22.09%	42.86	20.93%	51.15	8.14%	41.84
862	22.09%	42.24	22.09%	51.64	8.14%	41.50
733	22.09%	41.47	22.09%	52.21	9.30%	41.03
623	22.09%	41.55	23.26%	51.72	12.79%	40.07
530	22.09%	40.85	23.26%	51.28	11.63%	39.71
450	22.09%	40.40	23.26%	50.59	10.47%	39.20
382	22.09%	39.08	23.26%	50.70	9.30%	38.34
325	22.09%	38.62	23.26%	50.77	11.63%	37.43
276	22.09%	38.52	24.42%	49.21	9.30%	36.62
235	22.09%	38.71	24.42%	48.27	11.63%	36.06
200	22.09%	37.28	24.42%	47.67	12.79%	35.59
170	22.09%	36.33	24.42%	45.84	12.79%	35.13
144	22.09%	36.19	23.26%	44.64	15.12%	33.88
122	24.42%	35.43	24.42%	42.81	15.12%	33.48
104	24.42%	34.30	24.42%	40.87	15.12%	32.59
88	24.42%	32.87	29.07%	39.02	16.28%	31.99
75	24.42%	31.28	26.74%	36.56	17.44%	31.92
64	26.74%	29.84	25.58%	34.08	16.28%	31.80
54	26.74%	27.41	25.58%	30.07	17.44%	31.97
46	26.74%	25.94	26.74%	27.50	15.12%	31.90
39	25.58%	24.42	25.58%	25.10	12.79%	32.30
33	23.26%	22.53	29.07%	23.09	11.63%	32.66
28	20.93%	20.42	26.74%	19.87	15.12%	32.97
24	20.93%	18.86	24.42%	19.31	13.95%	33.16
20	23.26%	16.94	25.58%	16.59	16.28%	33.51
17	23.26%	15.83	24.42%	15.71	16.28%	33.58
14	22.09%	16.42	23.26%	16.15	17.44%	34.08
12	24.42%	17.72	26.74%	17.03	16.28%	34.55
10	25.58%	19.76	26.74%	19.95	13.95%	35.15
8	24.42%	22.45	29.07%	22.60	15.12%	36.01
7	24.42%	23.64	27.91%	24.16	16.28%	36.44
6	29.07%	25.35	27.91%	25.97	17.44%	36.87
5	27.91%	27.86	30.23%	28.73	18.60%	37.50

Num. of genes: the number of selected genes in the recursive steps. CV2: total cross-validation error rate (CV2 error rate). Mean SV: mean number of support vectors used in the cross-validation at each level.

**Table 10:** Selected biomarkers on the lung cancer dataset.

Marker	Rank	Gene title	Gene symbol	*t*-statistics	*P* value
D13705_s_at	1	Cytochrome P450, family 4, subfamily A, polypeptide 11	CYP4A11	5.743	9.52*E*−04
HG1103-HT1103_at	2	—	—	−5.434	1.77*E*−03
X07618_s_at	5	Cytochrome P450, family 2, subfamily D, polypeptide 6	CYP2D6	4.785	9.89*E*−03
Z71460_at	9	ATPase, H+ transporting, lysosomal V0 subunit a1	ATP6V0A1	4.644	0.0124
AFFX-HUMGAPDH/M33197_M_at	10	Glyceraldehyde-3-phosphate dehydrogenase	GAPDH	−4.496	0.0131
M37583_at	13	H2A histone family, member Z	H2AFZ	−4.445	0.0132
AFFX-HUMGAPDH/M33197_5_at	19	Glyceraldehyde-3-phosphate dehydrogenase	GAPDH	−4.161	0.0153
AFFX-HUMRGE/M10098_3_at	20	—	—	4.098	0.0153
D13748_at	22	Eukaryotic translation initiation factor 4A, isoform 1	EIF4A1	−4.245	0.0153
HG2279-HT2375_at	23	—	—	−4.099	0.0153
L14922_at	26	Replication factor C (activator 1) 1, 145 kDa	RFC1	−4.138	0.0153
U37143_at	35	Cytochrome P450, family 2, subfamily J, polypeptide 2	CYP2J2	4.245	0.0153
HG1153-HT1153_at	48	—	—	−4.031	0.0157
HG651-HT5209_s_at	49	—	—	3.995	0.0157
X01677_f_at	60	Glyceraldehyde-3-phosphate dehydrogenase	GAPDH	−4.008	0.0157
U25433_at	65	CATR tumorigenicity conversion 1	CATR1	3.971	0.0161
U38864_at	70	Zinc finger protein 212	ZNF212	3.941	0.0166
X77584_at	100	Thioredoxin	TXN	−3.807	0.0186
AFFX-HUMGAPDH /M33197_3_at	101	Glyceraldehyde-3-phosphate dehydrogenase	GAPDH	−3.774	0.0191
L34060_at	103	Cadherin 8, type 2	CDH8	3.789	0.0191
U96769_rna1_at	108	Chondroadherin	CHAD	3.782	0.0191
HG2855-HT2995_at	122	—	—	−3.714	0.0205
U66617_at	128	SWI/SNF related, matrix associated, actin dependent regulator of chromatin, subfamily d, member 1	SMARCD1	3.706	0.0205
U77970_at	191	Neuronal PAS domain protein 2	NPAS2	3.544	0.0237
L25085_at	195	Sec61 beta subunit	SEC61B	−3.535	0.024
D45248_at	243	Proteasome (prosome, macropain) activator subunit 2 (PA28 beta)	PSME2	−3.407	0.0285
U55764_at	273	Sulfotransferase family 1E, estrogen-preferring, member 1	SULT1E1	3.359	0.0303
J03258_at	295	Vitamin D (1,25-dihydroxyvitamin D3) receptor	VDR	−3.314	0.0316
L19067_at	430	v-rel reticuloendotheliosis viral oncogene homolog A, nuclear factor of kappa light polypeptide gene enhancer in B-cells 3, p65 (avian)	RELA	−3.106	0.0424
D38583_at	433	S100 calcium binding protein A11	S100A11	−3.098	0.0431
HG2815-HT2931_at	475	—	—	−3.039	0.0469
U20350_at	610	Chemokine (C-X3-C motif) receptor 1	CX3CR1	2.891	0.0567
U08815_at	772	Splicing factor 3a, subunit 3, 60 kDa	SF3A3	2.743	0.0683

Rank: gene-ranking is computed using *P* values.
